# Exploring the Potential of GaN-Based Power HEMTs with Coherent Channel †

**DOI:** 10.3390/mi14112041

**Published:** 2023-10-31

**Authors:** Xinghuan Chen, Fangzhou Wang, Zeheng Wang, Jing-Kai Huang

**Affiliations:** 1 The China Electronic Product Reliability and Environmental Testing Research Institute, Guangzhou 510610, China; 2 Songshan Lake Materials Laboratory, Dongguan 523808, China; 3 Manufacturing, CSIRO, Lindfield, NSW 2070, Australia; 4 Department of Systems Engineering, City University of Hong Kong, Hong Kong, China; 5 School of Materials Science and Engineering, University of New South Wales (UNSW), Sydney, NSW 2052, Australia

**Keywords:** GaN, HEMT, figure of merit, coherent channel

## Abstract

The GaN industry always demands further improvement in the power transport capability of GaN-based high-energy mobility transistors (HEMT). This paper presents a novel enhancement-type GaN HEMT with high power transmission capability, which utilizes a coherent channel that can form a three-dimensional electron sea. The proposed device is investigated using the Silvaco simulation tool, which has been calibrated against experimental data. Numerical simulations prove that the proposed device has a very high on-state current above 3 A/mm, while the breakdown voltage (above 800 V) is not significantly affected. The calculated Johnson’s and Baliga’s figure-of-merits highlight the promise of using such a coherent channel for enhancing the performance of GaN HEMTs in power electronics applications.

## 1. Introduction

In recent years, wide bandgap semiconductor devices have gained significant attention in power electronics due to their superior performance compared to their silicon-based counterparts [1,2,3,4]. Among these, Gallium Nitride (GaN) and its alloys with Indium and Aluminum have been shown to be particularly promising for high-frequency applications, thanks to the high mobility two-dimensional electron gas (2DEG) that forms in their heterojunctions [5,6,7,8,9,10]. The AlGaN/GaN High Electron Mobility Transistor (HEMT) is one such device that takes advantage of this high mobility 2DEG, with electron mobilities that can exceed 1800 cm^2^/V·s [11,12].

However, natural GaN HEMTs are depletion-mode devices due to their intrinsically continuous 2DEG, which can lead to standby leakage and increase the complexity of driver circuits [13,14,15], therefore, the enhancement-mode devives are desired in power electronic applications. Various device structures, such as fluoride ion implantation, pillar structure, and field coupling gate, have been proposed and explored to address this issue [16,17,18]. Additionally, extensive studies from our group and others have demonstrated that p-GaN on HEMT is a charming strategy for the realization of the enhancement-type GaN HEMTs based on the process in contemporary foundries [19,20,21,22,23]. Despite these efforts, the low concentration of the thin 2DEG still limits the current transportation, preventing the device from reaching its full potential [24].

To overcome this limitation, we hereby explore the use of a coherent channel for realizing a novel AlGaN/GaN HEMT with an enhancement-type functionality and very high-power transmission capability based on p-GaN-on-HEMT architecture [25]. Our device uses a coherent channel consisting of a GaN cap channel with n-type doping and an AlGaN layer channel with graded Al mole fraction. The graded Al mole fraction broadens the conduction band and creates a three-dimensional electron sea (3DES), which is different from the electron slab induced by buck doping. This new structure allows for a significantly higher current density and a higher breakdown voltage (BV) compared to traditional HEMTs. A numerical analysis of our proposed device shows that it has the potential to boost the performance of GaN-based power applications significantly.

## 2. Structure and Mechanism

**Figure 1:**
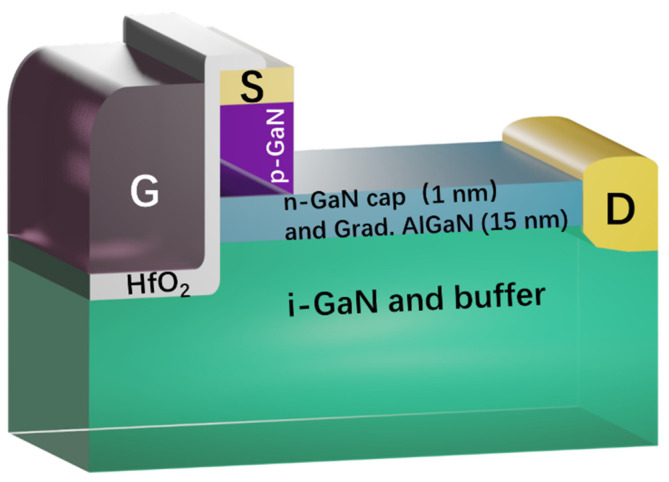
Rendered illustrations of the device structure.

**Figure 2:**
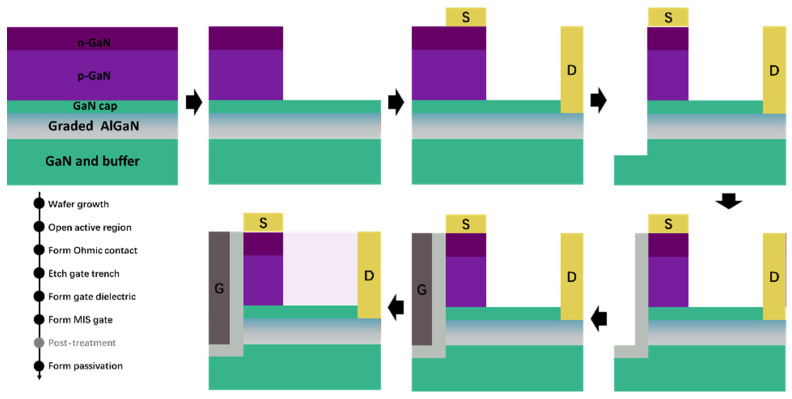
Schematical illustration of the fabrication process of the proposed device.

The proposed Coherent Channel High Electron Mobility field-effect Transistor (CC-HEMT) structure with three-dimensional electron sea (3DES) heterojunction can be fabricated on an AlGaN(graded)/GaN wafer, which can be realized by the typical MOCVD process. In this structure, the Al mole fraction of the AlGaN layer (15 nm) linearly increases from 0 at the heterojunction to 0.3 at the AlGaN surface, as shown in Figure 1. A p-type GaN layer is then deposited on top of the heterojunction, followed by a highly n-type-doped thin GaN layer of 10 nm, which serves as the active region of the source to ensure that the Ohmic contact is based on n-GaN for source and drain so that the source and drain Ohmic contact can be formed simultaneously. An etching and passivation process between the p-GaN pillar and drain electrode should be employed to prevent p-type leakage from the source to the drain by RIE/ICP-RIE and PECVD, and the passivation dielectric is Silicon Nitride (SiN). The gate is designed in a trench form and is covered with a 10 nm HfO_2_ dielectric layer, which can be conducted through ALD growth to form high quality gate dielectric. The gate metal extends over the source and 3DES regions, creating a continuous current channel perpendicular to the heterojunction. The standard fabrication process can be referred to as shown in Figure 2. The aforementioned processes have been well-developed and have already shown potential for achieving the superior performance of various GaN-based devices [26,27,28,29,30,31].

**Figure 3:**
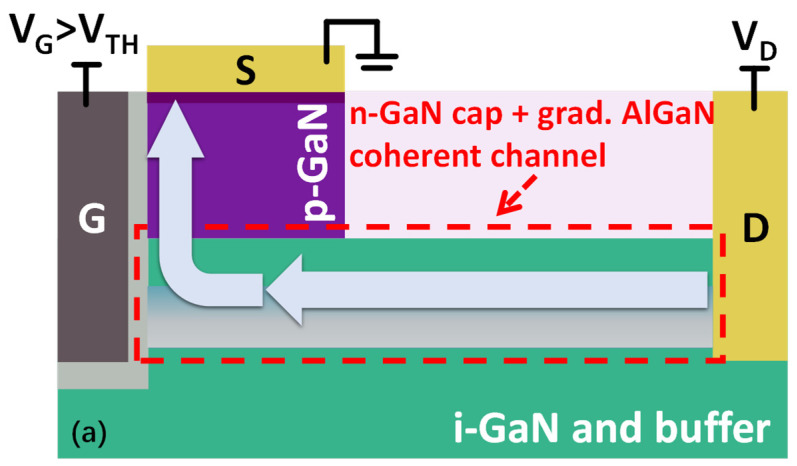

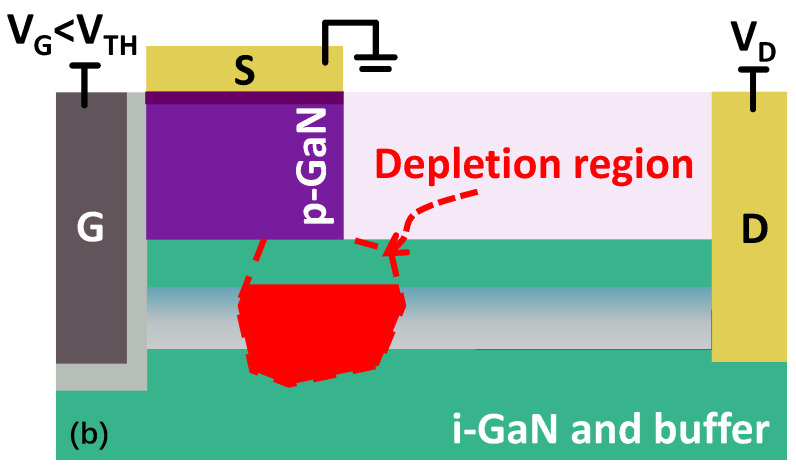
Schematical illustrations of the device’s working mechanisms under (**a**) turned-on and (**b**) blocking bias.

The working mechanisms of the proposed device under different biasing conditions can be seen in Figure 3. During off-state, the inversion channel is not formed in the p-GaN layer, so the Ohmic source and 3DES channel are separated by the p-GaN layer, and the device blocks high drain voltage by the reverse-biased p-GaN/3DES junction. When the device turns on, an inversion channel is formed in the p-GaN layer, connecting the 3DES and the Ohmic source, and current will flow though the coherent channel (combined by n-type GaN cap and graded AlGaN) and the inversion channel formed in p-GaN layer. The channel length is defined by the inversion layer, which should not be too short and located at a too heavily doped p-type region. A short channel may not be practically achievable due to fabrication process limitations or induce the short channel effect. On the other hand, a heavily doped p-GaN layer can cause depletion in the 3DES, leading to channel pinch-off. In this design, the gate-to-source length is set at 1 μm. Further device specifications are listed in Table 1. The Silvaco tool is used to simulate the device’s performance.

**Figure 4:**
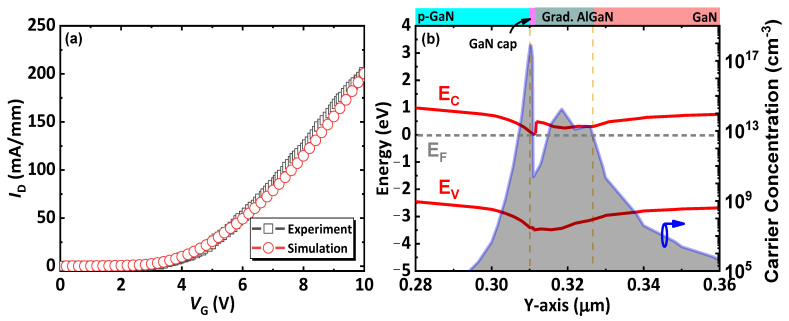
(**a**) The calibration of the simulation tool; (**b**) the simulated energy band diagrams and the three-dimensional electron sea distribution at the coherent channel.

Prior to simulation, a calibration of the physical models used in this work is performed. The calibrated device is chosen as a p-GaN gate HEMT due the he similar material composition as the proposed CC-HEMT, such as p-GaN, AlGaN and i-GaN. And the simulated data fits well with the experimental data from the same HEMT device structure with a p-GaN gate [32], as shown in Figure 4a, indicating that the settings of physical models used in this work are reasonable. The detailed settings of the physics models, such as the Shockly–Read–Hall recombination model, Fermi–Dirac static model, electric field dependence model concentration dependent mobility model, and impact ionization model, are the same as our previous publications [16,18,33,34].

Table 1 shows the specifications of the simulated device in this work, including the short name, full name, and the doping concentration and dimensional values.

## 3. Results and Discussion

Figure 4b displays the energy band structure of the proposed CC-HEMT perpendicular to the wafer through the p-GaN pillar. The graded Al fraction lowers the energy band, resulting in the formation of 3DES with high electron density above 10^13^ cm^−3^ in the AlGaN layer, which means the conduction channel is expanded to be a coherent three-dimensional channel instead of the sheet conduction channel of 2DEG in conventional GaN HEMTs. However, since the p-GaN partially depletes the electrons, the Fermi level above the band gap for p-GaN in the p-GaN layer depletion region, and the 3DES region does not span the entire AlGaN layer, as shown in Figure 4b, where the simulated 3DES length is approximately 25 nm. Additionally, the doping and polarization of the thin GaN cap layer contribute to a peak concentration of electrons located near the interface. Although, due to polarization, a valley of the concentration appears at the top of the coherent channel, the lowest concentration of the valley is still higher than 10^10^ cm^−3^. The formation of 3DES approximately aligns with recent experiments [35] and theoretical calculations [24].

**Figure 5:**
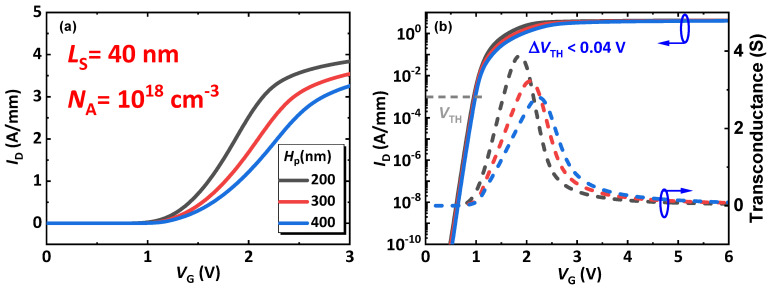
The transfer performance of the device in the (**a**) linear coordinator and (**b**) semi-log coordinator (with transconductance).

Figure 5a,b represents the transfer performance of the CC-HEMT. A narrow source of 40 nm (L_S_) is utilized as an example to improve simulation efficiency. Furthermore, to verify the functionality of the proposed device, the p-GaN doping limit of 10^18^ cm^−3^ is utilized, reflecting current fabrication processes and making for a challenging condition simulation to explore the device performance’s boundary. As indicated in the figure, the current transportation capability of the device decreases as the p-GaN height (H_P_) increases; this is due to the equivalent increase in the resistance of the channel. Nonetheless, the current flowing through the device is over 3 A/mm in all three samples when the drain voltage is 6 V, which is much higher than the calibrated p-GaN gate HEMT. This high performance is attributable to the high density of 3DES formed in the graded AlGaN layer, which is the key feature of the CC-HEMT. As shown in Figure 5b, threshold voltage (V_TH_, defined at I_D_ is 1 mA/mm) is insensitive to H_P_, as H_P_ increases from 200 nm to 400 nm, the varation of V_TH_ is lower than 0.04 V, which means the proposed CC-HEMT has a lager process tolerance. Also, shorter *H*_P_ results in higher peak transconductance—This is because of the merit of higher gate controllability through shorter channel length. For the pinch-off region (V_G_ < 1V), H_P_ does not influence the leakage significantly, mainly because of the stable depletion region of the p-n junction formed by p-GaN and the coherent channel, which does not extend to exceed 200 nm. It should be noted that this paper aims at exploring the use of a coherent channel for realizing a novel AlGaN/GaN HEMT with an enhancement-type functionality with very high-power transmission capability based on p-GaN-on-HEMT architecture and the device performance’s boundary, so the comparation between the calibrated device and the proposed device is not performed. 

However, it should be noted that the maximum Al content and thickness of the AlGaN layer should be traded off while considering the p-GaN doping concentration. High p-type doping may completely deplete the 3DES, which could be prevented by increasing the Al gradation of the AlGaN layer. However, in doing so, the resistance of the layer will also increase, resulting in a reduction of the device’s current transportation performance. Consequently, the optimal device configurations should be studied further while considering the aforementioned factors.

**Figure 6:**
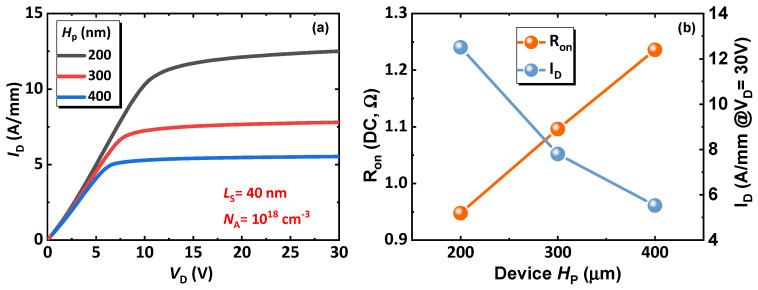
(**a**) Output performance of the proposed device with varying p-GaN heights, and (**b**) the key performance indicators extracted from (**a**).

Figure 6 gives the output performance of the proposed CC-HEMT with various H_P_ from 200 nm to 400 nm under V_GS_ of 4 V, where the output curves exhibit good saturation performance, indicating that the channel is resilient to parasitic effects like the short-channel effect. Compared to other recently reported devices, the proposed device boasts a very high current transmission capability [18,22,34,36]. Because of higher series resistance, higher p-GaN thickness turns out to be higher DC R_ON_, as can be seen in the Figure 6b—the device saturation current drops as a consequence. However, due to the high electron density of 3DES, the lowest saturation current with H_P_ of 400 nm still stays higher than 5 A/mm, which suggests the proposed device exhibits the desired high potential in power applications.

**Figure 7:**
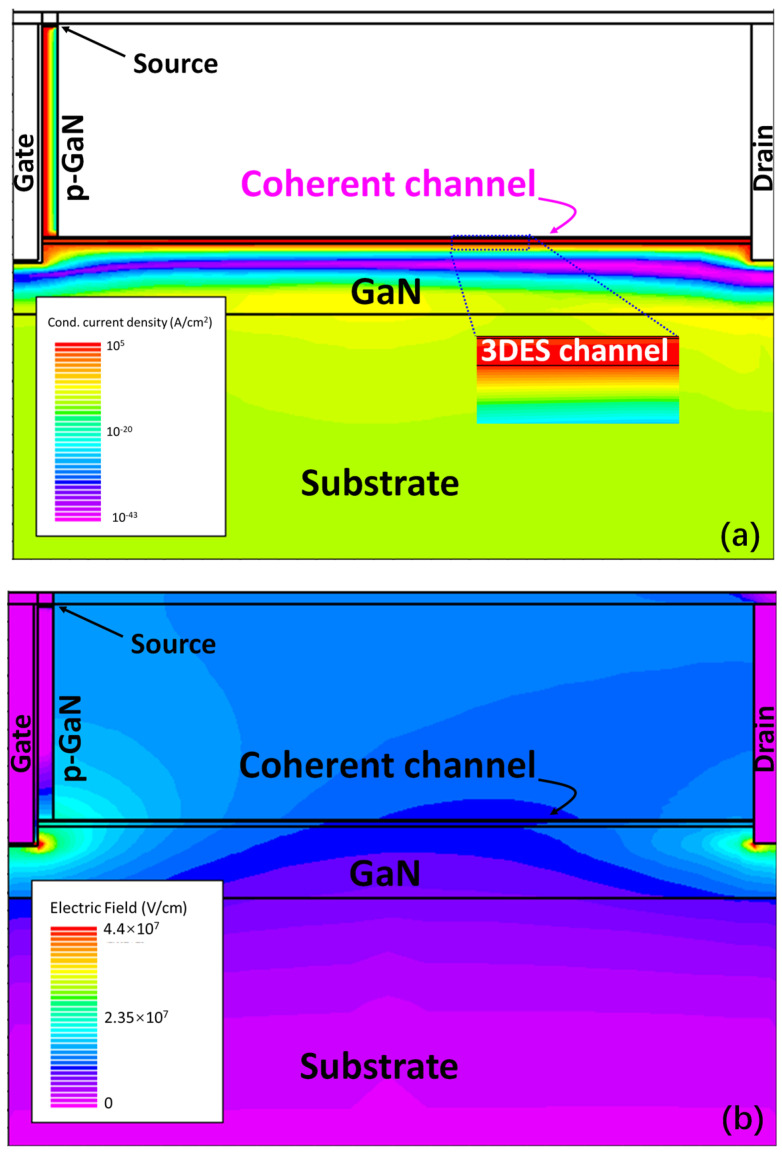
Simulated (**a**) current density in a forward-biased device and (**b**) electric-field distribution in a blocking device. Note that these two figures are from devices with different geometric specifications in order to achieve the best contrast.

Figure 7 presents the simulated results of the current density and electric field distribution of the proposed device working in a forwarding or blocking state. It can be seen from Figure 7a that an inversion channel is formed in the p-GaN layer to connect the 3DES and the Ohmic source and the main part of the current flows just through the coherent channel (the coherent channel is highlighted by a zoom out insert) when the device turns on (V_G_ = 4V and V_D_ = 3V), which indicates that the high current transportation capability is attributable to the high density of 3DES formed in the graded AlGaN layer. Also, the reverse blocking, as in Figure 7b, shows the same behavior as the mechanism of the design—The electric field crowded under the p-GaN pillar (V_D_ = 800V and other gates are grounded). Meanwhile, the stable depletion region of the p-n junction formed by p-GaN and the coherent channel can reduce the leakage significantly. It is expected that the depletion region of the device can extend towards the drain within the buffer layer, where the material’s critical electric field is relatively high so that a higher breakdown voltage can be obtained.

**Figure 8:**
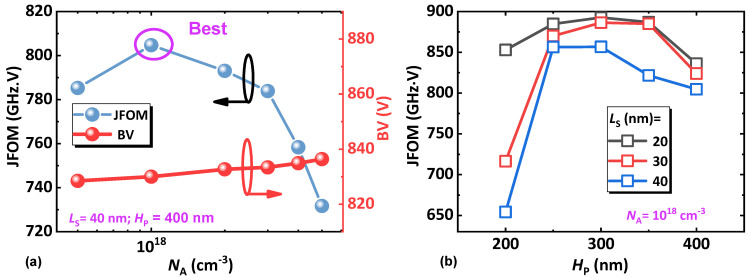
Figure-Of-Merit (FOM) of the proposed device with different (**a**) doping and (**b**) height of p-GaN pillar.

Figure 8 shows Johnson’s Figure-Of-Merit (JFOM) of the proposed device with different specifications of the p-GaN pillar. For the JFOM, it is related to the saturation electron velocity of the device $V_{sat}$ and the critical electric field $E_{C}$, as below [37,38]:

(1) $$JFOM=\frac{V_{sat}E_{C}}{2\pi }$$

The $V_{sat}$ is dependent on the cut-off frequency $f_{T}$ and the length of the gate $L_{G}$ (in our case, this should equal the height of the p-GaN, namely the length between the source and the coherent channel):

(2) $$V_{sat}=f_{T}\cdot 2\pi \cdot L_{G}$$

We can approximately estimate the $E_{C}$ by using the breakdown voltage BV and introducing a linear component of fitting, a:

(3) $$E_{C}=\frac{2BV}{a\cdot L_{G}}$$

Therefore, if the adjustable parameter is 2, the frequency JFOM can be obtained in a form of:

(4) $$JFOM=f_{T}\cdot BV$$

As can be seen in Figure 8, the device features high JFOM values; this can be attributed to the functionality of the coherent channel, where the high-density, large-volume 3DES is formed by the combination of doping (for the n-GaN cap layer) and polarization (for the graded AlGaN layer). Before the best point of doping concentration, higher doping yields higher JFOM; this is because the depletion region between the p-GaN and the coherent channel decreases, and this decreasing trend dominates the JFOM. The concentration exceeding the best point, however, will reduce the mobility of the vertical channel inside the p-GaN, which starts to dominate in lowering the JFOM.

Figure 8b can be achieved by fixing the p-GaN doping to 10^18^ cm^−3^, varying H_P_ from 200 nm to 400 nm and varing L_S_ from 20 nm to 40 nm. In this figure, it can be seen that the best height of the p-GaN is around 300 nm for the high JFOM. Lower heights can reduce the BV and, therefore, reduce the JFOM, while higher heights can lower the channel conductivity, as shom in Figure 6b, and result in a lower cut-off frequency. These two factors need to be considered in further studies.

**Figure 9:**
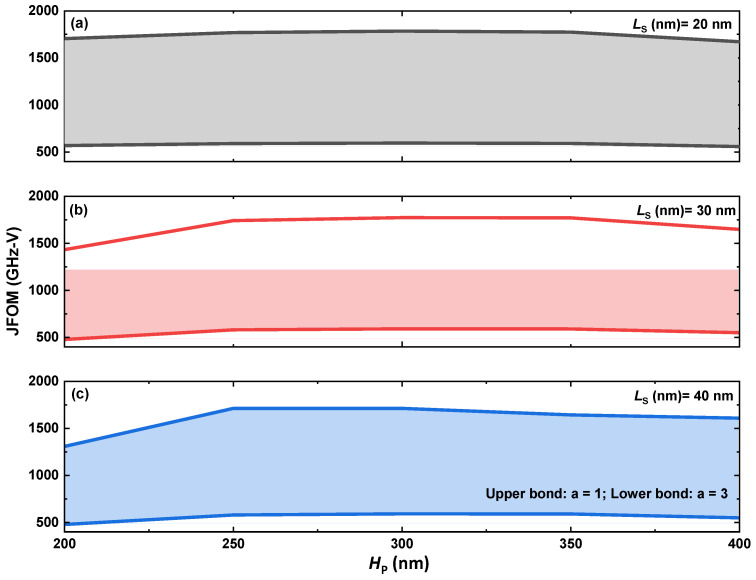
The performance boundary of JFOM when the adjustable parameter varies from 1 to 3. (**a**) L_S_ of 20 nm. (**b**) L_S_ of 30 nm. (**c**) L_S_ of 40 nm.

**Figure 10:**
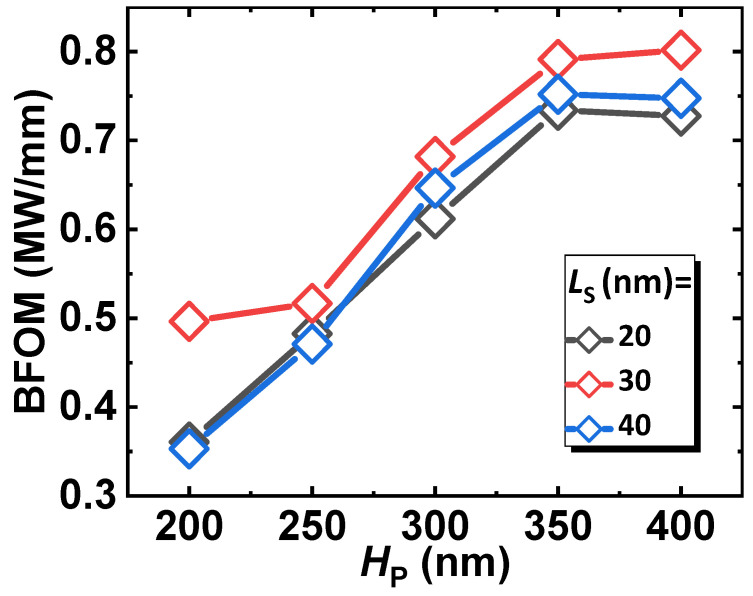
The Baliga’s FOM of the proposed device with different specifications.

If considering the adjustable factor a in Equation (3), a more comprehensive trend of JFOM vs. H_P_ can be drawn with various L_S_ from 20 nm to 40 nm, as in Figure 9. According to the simulation, a THz-level JFOM can be obtained in the best cases. This high performance is the direct consequence of the feat of the coherent channel. In this channel, the polarization layer provides the high-mobility component of the coherent channel, while the doping layer provides extra carriers for current transport. With such a combination, the coherent channel can exhibit high JFOM as well as a high Baliga’s FOM (BFOM), as can be seen in Figure 10.

In particular, the BFOM peaks when the height of the p-GaN reaches around 350 nm; this indicates that the breakdown happens within the p-GaN until it is higher than 350 nm—then the breakdown is the responsibility of the coherent channel. Therefore, we can achieve an even higher BFOM, with high JFOM remaining, by extending the coherent channel length. All these facts suggest that the proposed architecture of CC-HEMT with a coherent channel can be favored in future power applications.

It should also be noted that this research is a proof-of-concept study, and some of the parameters adopted here are ideal. In reality, owing to the limits of the fabrication process, the presence of traps and defects may significantly influence the final performance of the device. Further studies are required to validate the superiority of the proposed device experimentally, which is not the scope of the current study.

## 4. Conclusions

In conclusion, the proposed CC-HEMT demonstrates outstanding high-power performance, which is attributed to the introduction of the coherent channel by the graded AlGaN layer with the n-GaN cap layer. The graded Al fraction lowers the energy band, resulting in the formation of 3DES with high electron density above 10^13^ cm^−3^ in the AlGaN layer. The device exhibits a remarkable on-state current exceeding 3 A/mm and a high BV of over 800 V, which suggests the proposed device exhibits the desired high potential in power applications. Meanwhile, the proposed CC-HEMT can achieve an even higher BFOM, with high JFOM remaining, by extending the coherent channel length. And the Although further optimization of the device configuration is necessary, the CC-HEMT holds great potential in enhancing the overall performance of future power applications, such as LED power management, wireless power transmission, and charging stations, according to a rigorous numerical analysis presented.

## Figures and Tables

**Table 1:** Specifications of the simulated device in this work.

Short Name	Full Name	Value
*L*_S_	Source length	40 nm
*H*_P_	p-GaN height	200 to 400 nm
*N*_A_	p-GaN doping concentration	10^18^ cm^−3^
*/*	n-GaN doping concentration	3 × 10^20^ cm^−3^
*/*	GaN cap doping concentration	2 × 10^20^ cm^−3^
*/*	Thickness of n-GaN	10 nm
*/*	Thickness of GaN cap	1 nm
*/*	The thickness of graded AlGaN	15 nm

## Data Availability

All data in this study can be accessed upon reasonable request to the corresponding authors.
